# Neutrophils in cancer carcinogenesis and metastasis

**DOI:** 10.1186/s13045-021-01187-y

**Published:** 2021-10-21

**Authors:** Shumin Xiong, Liaoliao Dong, Lin Cheng

**Affiliations:** Shanghai Institute of Hematology, State Key Laboratory of Medical Genomics, National Research Center for Translational Medicine at Shanghai, Ruijin Hospital Affiliated to Shanghai Jiao Tong University School of Medicine, Shanghai, 200025 China

**Keywords:** Neutrophil, Cancer, Microenvironment, Cell plasticity, Cell reprogramming

## Abstract

In recent years, neutrophils have attracted increasing attention because of their cancer-promoting effects. An elevated neutrophil-to-lymphocyte ratio is considered a prognostic indicator for patients with cancer. Neutrophils are no longer regarded as innate immune cells with a single function, let alone bystanders in the pathological process of cancer. Their diversity and plasticity are being increasingly recognized. This review summarizes previous studies assessing the roles and mechanisms of neutrophils in cancer initiation, progression, metastasis and relapse. Although the findings are controversial, the fact that neutrophils play a dual role in promoting and suppressing cancer is undeniable. The plasticity of neutrophils allows them to adapt to different cancer microenvironments and exert different effects on cancer. Given the findings from our own research, we propose a reasonable hypothesis that neutrophils may be reprogrammed into a cancer-promoting state in the cancer microenvironment. This new perspective indicates that neutrophil reprogramming in the course of cancer treatment is a problem worthy of attention. Preventing or reversing the reprogramming of neutrophils may be a potential strategy for adjuvant cancer therapy.

## Background

Neutrophils have been recognized as the most abundant innate immune cells in both bone marrow and peripheral blood [1]. They are rapidly recruited into sterile or infected inflammation sites and show high plasticity and a strong effector response. Perhaps to avoid unnecessary tissue damage, neutrophils possess a short lifespan [2]. Therefore, the abundance of neutrophils relies on constant replenishment via granulopoiesis in the bone marrow. Their origin is hematopoietic stem cells, which give rise to lymphoid-primed multipotent progenitors (LMPPs). Neutrophils are derived from the early committed neutrophil progenitor (proNeu1), a subtype of granulocyte–monocyte myeloid progenitor (GMP) that develops from LMPPs [3, 4]. Classically, as determined based on nuclear morphology, neutrophils mature through the following sequence: GMPs, myeloblasts, promyelocytes, myelocytes, metamyelocytes, banded neutrophils and segmented neutrophils [1]. According to recent studies, the neutrophil developmental pathway mapped based on single-cell analyses is proNeu1, intermediate progeny (proNeu2), preneutrophil (preNeu), immature neutrophils and, finally, mature neutrophils [4]. Transcription factors, such as C/EBPα and C/EBPε, exquisitely control neutrophil development [5–7]. During neutrophil maturation, migration and immune response functions gradually overtake proliferation. Both microbial and cancer stresses trigger preNeu expansion and immature neutrophil release from bone marrow [8]. Moreover, extramedullary granulopoiesis commonly occurs in the spleen under pathological states [9].

Neutrophils play various roles in different diseases, including infectious diseases, metabolic diseases, autoimmune diseases and aging-associated diseases. On the one hand, neutrophils exert positive functions in host defense, including antibacterial [10], antifungal [11] and antiviral [12] functions. In addition, they eliminate apoptotic cell debris, which is beneficial for tissue regeneration and angiogenesis after tissue damage [13]. On the other hand, neutrophils are involved in pathogenesis through diverse mechanisms. First, neutrophils recruited to the lesion site release proteases and produce a large amount of reactive oxygen species (ROS), resulting in tissue damage, rendering the tissue more susceptible to pathogens and even the development of chronic inflammation [14]. This pathological effect on many infectious diseases and pulmonary diseases, including severe cases of coronavirus disease 2019 (COVID-19), has frequently been observed [15]. In addition, neutrophil elastase (NE) causes insulin resistance during the development of obesity and type 2 diabetes [16]. Second, neutrophils may shift their function to immunosuppression characterized by a lower response to chemokines and inhibition of T cell immunity. In sepsis, this functional change is life-threatening [17]. Third, neutrophil extracellular traps (NETs) extruded by activated neutrophils have been reported to participate in the occurrence and development of a wide range of diseases. NETs are large extracellular complexes composed of cytosolic and granule proteins and chromatin [18]. In individuals with atherosclerosis, NETs result in the destabilization of atherosclerotic plaques through the lysis of smooth muscle cells [19]. NETs are also the major inducers of thrombosis [20]. In autoimmune diseases, such as systemic lupus erythematosus, rheumatoid arthritis and ANCA-associated vasculitis, NETs are recognized as antigens that contribute to the production of anti-self-antibodies [21]. In general, neutrophils are a double-edged sword in diseases with both defensive and harmful functions.

Neutrophils, the most dominant immune cells [22], also play complex and important roles in cancer. Many studies have reported elevated peripheral blood counts of neutrophils in patients with different cancers. The neutrophil-to-lymphocyte ratio (NLR) has been shown to be an independent prognostic indicators for patients with cancer [23]. This review will describe the multifaceted roles of neutrophils in cancer initiation, growth and metastasis, thereby revealing the heterogeneity and high plasticity of neutrophils in cancer. Based on these findings and those from our own studies, we attempted to analyze the possible mechanism of neutrophil heterogeneity from the perspective of cell reprogramming.

## Neutrophils in carcinogenesis

### Cancer initiation

Inflammation plays an essential role in cancer initiation by damaging tissues, and neutrophils are a crucial component of this process. Thus, neutrophils provide a link between inflammation and cancer. Cancer that develops in various mouse models of KRAS-driven ovarian cancer exhibits upregulated levels of neutrophil-related chemokines and an expansion of neutrophils. These phenotypes may result from direct upregulation of neutrophil-related cytokines such as GM-CSF and CXCL8 [24, 25]. In a zebrafish model of HRAS^G12V^-driven melanoma, wounding-induced inflammation with elevated levels of prostaglandin E2 increase the formation of cancer in a neutrophil-dependent manner [26]. Depletion of the entire neutrophil population using anti-Ly6G antibodies impairs carcinogenesis in both chemically induced and spontaneous cancer models. Neutrophils overexpressing CXCR2 are attracted to cancer-prone tissues via the cytokine IL-8 and chemokine ligands CXCL1, CXCL2 and CXCL5. The application of chemical carcinogens in CXCR2-deficient mice, which show impaired neutrophil trafficking, prevents papilloma or adenoma formation [27, 28]. CXCR2-mediated neutrophil trafficking from bone marrow into peripheral blood is antagonized by CXCR4 expression due to the retention of neutrophils by CXCL12-expressing bone marrow stromal cells mediated retention. Bone marrow macrophages subsequently eliminate the retained neutrophils in a rhythmic manner.

### Neutrophils induce DNA damage

The evidence described above has indicated that neutrophils are crucial for carcinogenesis, but the exact mechanisms by which neutrophils foster carcinogenesis require further elucidation. Neutrophils produce and release genotoxic DNA substances that increase DNA instability. In an in vitro coculture model mimicking intestinal inflammation in ulcerative colitis, neutrophils increase errors in the replication of colon epithelial cells. In individuals with chronic colon inflammation, activated neutrophils cause an accumulation of target cells in G2/M phase, consistent with the installation of a DNA damage checkpoint [29]. Neutrophil-derived elastase, neutrophil production of ROS, reactive nitrogen species (RNS) and angiogenic factors such as MMP-9 and the immunosuppressive ability of neutrophils may be associated with this process. ROS released by neutrophils during chronic inflammation, such as hypochlorous acid (HOCl, formed by myeloperoxidase (MPO)), cause DNA damage and are mutagenic in lung cells in vitro. HOCl is a major neutrophil oxidant. MPO-catalyzed formation of HOCl during lung inflammation is an important source of neutrophil-induced genotoxicity. Neutrophils cause DNA damage by releasing ROS and inducing gene mutations in premalignant epithelial cells, thus driving oncogenic transformation in lung cancer. Additionally, at physiological concentrations, HOCl induces mutations in the hypoxanthine phosphoribosyl transferase (HPRT) gene, inducing three major types of DNA lesions [30]. Haqqani and coworkers analyzed a mouse model of subcutaneous cancer and showed that inducible nitric oxide synthase (iNOS) and nitric oxide synthase (NOS) contents and neutrophil infiltration were significantly correlated with the number of mutations in the *Hprt* locus [31]. However, a new mechanism that does not rely on ROS was also recently identified. In clinical samples from patients with inflammatory bowel disease and injury models, activated tissue-infiltrating neutrophils release particles carrying proinflammatory microRNAs, including miR-23a and miR-155, which increase DNA double-strand breaks and genomic instability [32]. miR-155 is also responsible for neutrophils-induced DNA damage and DNA repair landscape in acute colon injury, resulting in colorectal cancer initiation even shaping the progression [33].

### Neutrophils promote angiogenesis and immunosuppression

Coussens et al. documented that MMP-9 supplied by bone marrow-derived neutrophils and other hematopoietic cells contributes to squamous carcinogenesis [34]. MMP-9 produced by neutrophils also contributes to the carcinogenesis of pancreatic islet carcinoma and lung cancer accelerating angiogenesis [35]. NETs promote inflammation in subjects with nonalcoholic steatohepatitis, resulting in the development of hepatocellular carcinoma, which is inhibited by deoxyribonuclease treatment or peptidyl arginine deaminase type IV knockout, decreasing NET formation [36]. Furthermore, NETs positively correlate with the increased number of regulatory T cells (Tregs) in cancer by facilitating naïve CD4^+^ T cell metabolic reprogramming. Therapies targeting the interaction between these two cell types or inhibiting Treg activity may promote cancer immunosurveillance and prevent hepatocellular carcinoma formation [37].

In summary, neutrophils recruited to inflammatory sites promote cancer initiation mainly by increasing DNA damage, angiogenesis and immunosuppression. However, the mechanism underlying neutrophil-dependent carcinogenesis is complicated and cannot be reduced to one specific molecule. Even the same molecule often exerts different effects on diverse stages. Although CXCR2 promotes neutrophil migration into pro-cancer sites, knockdown of CXCR2 in neutrophils increases ROS production and exerts pro-cancer effect [38]. Thus, in future studies, genetically engineered mouse models (GEMMs) will be extremely valuable for research in the field of cancer-related neutrophil biology, as they enable neutrophils and neutrophil-derived factors to be manipulated as cancer arises de novo.

## Neutrophils in cancer progression

More than two decades ago, neutrophils were presumed to cause cancer xenograft rejection in mice [39, 40]. Just a few years later, the opposite result was reported: depletion of neutrophils reduced the growth of transplanted cancer [41]. Since then, reports of neutrophils promoting cancer progression have vastly outpaced those of neutrophils inhibiting cancer.

### Neutrophils promote cancer growth

The mechanisms by which neutrophils promote cancer growth are diverse. Neutrophils are characterized by rich granules, which perform different functions (Table 1). Some granule proteins (MMP-9 and ARG-1) released by activated neutrophils are associated with cancer progression. For example, MMP-9 released by neutrophils degrades the extracellular matrix, which in turn releases vascular endothelial growth factor (VEGF) and promotes angiogenesis [42]. Depletion of neutrophils or blockade of CXCR2 signaling to affect neutrophil recruitment inhibits cancer growth and reduces angiogenesis [43]. In contrast, an injection of cancer cells with neutrophils from cancer-bearing mice increases cancer growth and angiogenesis. In addition, the release of ARG-1 from neutrophils depletes arginine in T cells, causing the downregulation of CD3ζ. This process inhibits CD3-mediated T cell activation and proliferation, creating an immunosuppressive environment that also contributes to cancer growth [44]. In addition, the H^+^-pumping ATPase on tertiary granules causes cancer acidosis when it is mobilized to the cell surface, which may lead to cancer progression. Furthermore, an acidic pH inhibits the anticancer activity of T cells and natural killer (NK) cells, resulting in immune escape. Neutrophils also promote cancer growth and progression by recruiting macrophages and Tregs [45]. The structure of NETs formed by granule proteins and DNA induces the proliferation of cancer cells through high mobility group protein B1 (HMGB1) and NE [46–48]. In hematological malignancies, levels of NETs are found to positively correlated with lymphoma progression or childhood acute leukemia development [49, 50].

**Table 1:** The function of neutrophil proteins in cancer

Granule	Gene name	Protein name	Functions	References
Azurophil (primary) neutrophil granules	*AZU1*	Azurocidin	Antibacterial activity (Gram-bacteria); monocyte and fibroblast-specific chemotaxis; binds heparin; reprograms stellate cells toward a phenotype affecting the cancer microarchitecture; disrupts vascular endothelial cell morphology	[51–55]
	*DEFA1*-*4*	Neutrophil defensins	Antibacterial, fungicidal, and antiviral activities; enhance anticancer immunity; direct cytolysis(high concentration); induce apoptosis; inhibiting angiogenesis; stimulate cancer growth (low concentration); promotes invasiveness	[56–59]
	*PRTN3* (*MBN*)	Myeloblastin	Serine protease; facilitates transendothelial neutrophil migration; PRTN3-involved IκBα cleavage leads to abnormal activation of NFκB signaling pathway (carcinogenesis); inhibits T cell proliferation; mediates cancer metastasis to bone	[60–62]
	*CD63* (*MLA1*)	CD63 antigen	Cell surface receptor for TIMP1; induces NET formation; creates a premetastatic niche in the liver	[63, 64]
	*CTSG*	Cathepsin G	Antimicrobial, serine protease; facilitates neutrophil anti-cancer cytotoxicity; induces cell migration and multicellular aggregation; promotes metastasis; impairs NKp46-mediated responses of NK cells	[65–69]
	*ELA2* (*ELANE)*	Neutrophil elastase	Serine protease; facilitates primary cancer growth and secondary organ metastasis; selectively kills cancer cells and attenuates carcinogenesis; enhances cancer cell invasion; involved in awakening of dormant cancer cells; cleaves PML-RARα and is important for the development of APL in mice	[70–74]
	*MPO*	Myeloperoxidase	Microbicidal activity against a wide range of organisms; cancer cell cytotoxicity; awakening of dormant cancer cells by accumulation of oxidized lipids	[75, 76]
	*BPI*	Cap57; bactericidal permeability-increasing protein	Antibacterial, anticancer, and LPS-neutralizing activities; cancer cell cytotoxicity	[77]
Specific (secondary) neutrophil granules	*CHI3L1*	Chitinase-3-like protein 1	Glycoside hydrolase family 18; binds to chitin, heparin, and hyaluronic acid; plays a critical role in cancer cell growth, proliferation, invasion, metastasis, angiogenesis, activation of tumor-associated macrophages, and Th2 polarization of CD4 + T cells	[78]
	*NGAL* (*LCN2*)	Lipocalin 2	Antimicrobial; functions in innate immune defense; induces apoptosis of B lymphocytes; mediates appetite suppression; induces mesenchymal-epithelial transition of cancer cells thereby facilitating colonization and metastatic outgrowth	[79–81]
	*LTF* (*GIG12*)	Lactoferrin	Antimicrobial, anti-viral, antioxidant, anti-cancer, and anti-inflammatory activities; modulation of immune responses; anti-proliferation of cancer cell line; has a radiation resistance effect; LTF-IC can convert TAMs into M1-like cells	[82–85]
Gelatinase (tertiary) neutrophil granules	*MMP9* (*CLG4B*)	Matrix metalloproteinase-9	Contributions to squamous carcinogenesis; required for cancer vasculogenesis; promotes angiogenesis and cancer invasion; involved in awakening of dormant cancer cells	[34, 72, 86–88]
	*FCN1* (*FCNM*)	Ficolin-1	Pattern-recognition receptor in innate immunity; downregulated in cancer; not associated with cancer	[89–91]
	*CAMP*	Cathelicidin antimicrobial peptide	Antibacterial activity through binding LPS; cleaved into 2 antimicrobial peptides FALL-39 and LL-37; LL-37 plays a role in carcinogenesis; also displays anti-cancer effect	[92–95]
	*MMP8*	Neutrophil collagenase	Inhibiting NET formation; inhibits cancer cell invasion, proliferation and metastasis	[96, 97]
Other neutrophil proteins	*S100A8*/*A9*	S100-A8/A9	Proinflammatory protein; inflammation and oxidative stress; induces activation of myeloperoxidase; induces the release of gelatinase and specific granules; enhances cancer cell survival and chemoresistance; stimulates the recruitment of myeloid cells leading to cancer growth, formation of premetastatic niche, and metastasis	[76, 98]
	*ANXA1*	Annexin A1	Anti-inflammation protein; associated with cancer progression and metastasis; required in transvascular pumping of solid cancer; required in chemotherapy-induced anticancer immunity of dendritic cells; promotes immune cells infiltration	[99–103]

**Fig. 1:**
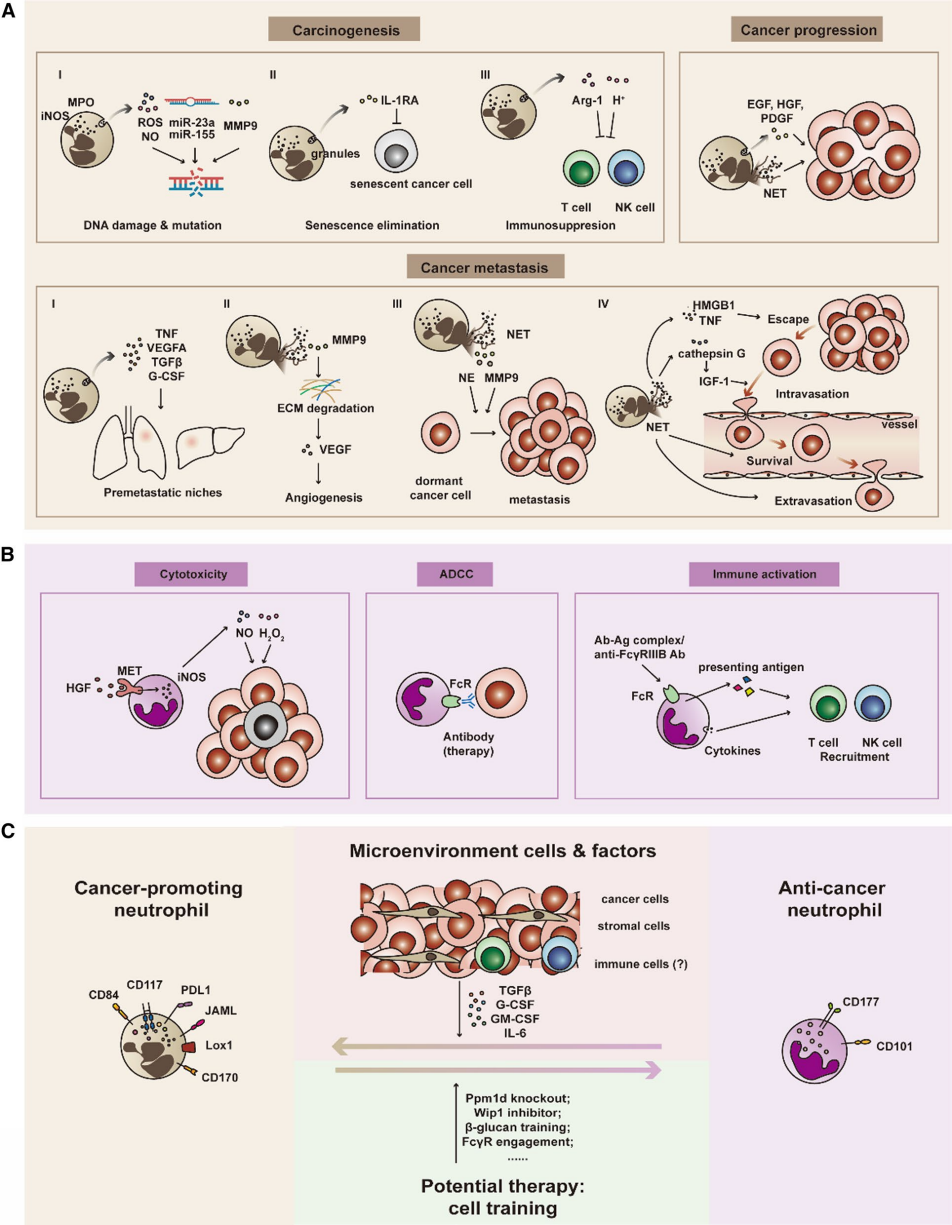
Dual roles and plasticity of neutrophils in cancer. **A** Neutrophils with cancer-promoting effects. Neutrophils promote cancer initiation, progression and metastasis: (1) Neutrophils cause DNA damage and gene mutation through ROS produced by MPO, NO produced by iNOS, microRNAs and MMP9, which induce carcinogenesis. (2) Neutrophils eliminate senescence through IL-1RA and thus promote cancer progression. (3) Immunosuppression mediated by the release of Arg-1 from neutrophils to inhibit CD3-mediated T cell activation and proliferation. An acidic pH inhibits the anticancer activity of T cells and NK cells. (4) The acidic pH, cytokines and NETs can increase cancer cell proliferation. (5) Neutrophils promote each step of cancer metastasis. Cytokines released by neutrophils prepare the premetastatic niche in distant organs. MMP9 induces angiogenesis by releasing VEGF from degraded ECM. HMGB1 and TNF promote the migration of cancer cells toward blood vessels. Cathepsin G promotes intravasation through the activation of IGF-1. NETs and the interaction between neutrophils and cancer cells promote cancer cell survival in the peripheral blood. NETs also facilitate extravasation. MMP9 and NE in NETs waken up dormant cancer cells in distant organs causing the formation of metastasis. **B** Neutrophils with anti-cancer effect. Neutrophils exert a cytotoxic effect via H_2_O_2_ and NO production induced by MET-mediated iNOS. ADCC during antibody therapy may be another mechanism by which neutrophils kill cancer cells. Chemokines produced by neutrophils recruit T cells and other leukocytes and indirectly kill cancer cells. **C** Reprogramming between protumor neutrophils and antitumor neutrophils. Generally, in the process of cancer progression, various cytokines released from cancer cells and stromal cells around them may transform anticancer neutrophils into protumor ones. Additionally, many experiments proved that protumor ones or normal neutrophils can be trained to function as anticancer neutrophils. The plasticity of neutrophils has been confirmed based on concrete evidence and should be considered in cancer therapy

In addition to granular proteins, neutrophils also play a role in promoting cancer growth by releasing growth factors, including epidermal growth factor, hepatocyte growth factor (HGF) and platelet-derived growth factor. Another study has shown that neutrophils eliminate senescence through IL-1 receptor antagonist (IL-1RA) and thus promote the progression of prostate cancer. Based on cancer promotion effect of neutrophil in pancreatic ductal adenocarcinoma (PDAC), lorlatinib inhibiting FES kinase, which is activated in neutrophils by PDAC cells, can attenuate cancer growth [104] (Fig. 1A).

### Neutrophils inhibit cancer growth

Although fewer studies have assessed the inhibitory effects of neutrophils on cancer, very interesting data have been reported. For example, in models transplanted with different cancer cell lines or spontaneous cancer models, changes in neutrophil recruitment induced by specifically knocking out neutrophil MET, the HGF receptor, increase cancer growth [105, 106]. In mice transplanted with mouse mammary cancer virus promoter-driven polyomavirus middle T antigen (MMTV-PyMT) or MMTV-myc mammary cancer, neutrophils may exert a cytotoxic effect by producing H_2_O_2_ and subsequently inhibit cancer growth. Antibody-dependent cellular cytotoxicity (ADCC) during antibody therapy may be another mechanism by which neutrophils kill cancer cells [107, 108]. Neutrophils express Fcγ receptors, which mediate cancer cell elimination through ADCC. Depletion of neutrophils reduces the efficacy of treatment with anti-CD52 mAb (alemtuzumab) and anti-CD20 mAb (rituximab) in mouse lymphomas [109]. IgA induces the killing of cancer cells by neutrophils much more strongly than IgG [110]. In addition, neutrophils slow cancer growth by controlling microbial populations and cancer-associated inflammation [111]. However, since endogenous antibodies usually activate the anticancer effects of neutrophils via Fc receptors, researchers have not determined whether ADCC occurs in vivo in the absence of exogenous antibodies (Fig. 1B).

Remarkably, many studies using the same transplanted cell lines reported the opposite results. This discrepancy may be caused by the use of different experimental methods or sampling times in each experiment. For example, the different antibodies used to deplete neutrophils have different corresponding targets and efficiencies. Neutrophils will have different functions in different stages of cancer progression and will gradually transform from exerting anticancer effects to producing cancer-promoting effects. All of these factors may have led to inconsistent conclusions. Therefore, future studies should focus on how the context affects neutrophil function.

## Neutrophils in cancer metastasis

In recent years, most studies examining the role of neutrophils in cancer have been related to metastasis. Combined intravenous injection of cancer cells and neutrophils from cancer-bearing rodents was found to increase the incidence of lung metastases as early as the late 1980s [112]. Subsequent studies have shown that the increased levels of neutrophils induced by the IL-17/G-CSF axis or the cholesterol metabolite 27-hydroxycholesterol promote cancer metastasis [113, 114], and the concentration of β2-integrin (CD18) in the intracellular granules of neutrophils is positively correlated with liver metastasis of colorectal cancer in mice [115]. Increased NETs also facilitate hepatocellular carcinoma cell metastasis by activating TLR4/9-COX2 signaling. NET-enabled metastatic activity is abrogated by inhibiting this signaling pathway [116]. Neutrophils are actively involved in each step of the metastatic cascade: formation of the premetastatic niche, cancer cell escape from the primary tumor, intravasation into the blood and/or the lymphatic vascular system, survival in the circulation, extravasation into distant organs, awakening of dormant cancer cells and outgrowth of metastases.

### Neutrophils promote cancer cell migration and intravasation

In the early stages of metastasis, neutrophils release MMP-9 to promote angiogenesis, playing an important role again by not only facilitating cancer growth but also providing more routes for cancer cells to escape. Neutrophils also direct cancer cells to endothelial cells, prompting them to enter the bloodstream. One mouse model of melanoma showed that cancer cells clustered around blood vessels and increased lung metastasis but had no effect on the growth of the primary tumor. In this model, cell damage increased HMGB1 levels, leading to the recruitment of neutrophils that subsequently promoted the migration of cancer cells toward blood vessels [117–119]. In vitro, neutrophil-derived tumor necrosis factor (TNF) stimulates melanoma cell migration, suggesting that TNF is one of the factors related to neutrophil-induced metastasis.

Next, neutrophils guide cancer cells into blood vessels. Cathepsin G, a neutrophil-derived serine protease, induces cell migration, activates insulin-like growth factor 1, increases E-cadherin-mediated intercellular adhesion and cancer cell aggregation, and promotes cancer cell entry into blood vessels [120]. NETs trap circulating cancer cells (CTCs), helping them spread to distant sites and promoting their adhesion to distant sites [121, 122]. The interaction between neutrophils and CTCs promotes cell cycle progression in the blood and expands the metastatic potential of CTCs [123]. According to a recent study, ROS produced by neutrophils increase NETs, especially in obese cancer-bearing mice, which weakens endothelial junctions and promotes the extravasation of cancer cells[124]. In addition, several studies have shown that direct interaction between neutrophils and cancer cells activates neutrophils, increases the migration of cancer cells, promotes the anchoring of cancer cells to endothelial cells, and ultimately helps cancer cells exit blood vessels [123, 125].

### Neutrophils facilitate cancer cell extravasation

Finally, metastatic cancer cells in distant tissues typically remain dormant for an extended period, during which infiltrating neutrophils release MMP-9 to promote angiogenesis, triggering the growth of dormant metastases. In addition, continued inflammation induces the formation of NETs, which are needed to wake dormant cancer cells. A mechanistic analysis has shown that two NEs and MMP-9, which are associated with NETs, cleave laminin. Cleaved laminin induces the proliferation of dormant cancer cells by activating α3β1-integrin signaling [72].

A related interesting phenomenon has been observed. Before disseminated cancer cells arrive, neutrophils accumulate in distant organs, forming the premetastatic niche. Neutrophils have been observed to aggregate in the lungs prior to the occurrence of metastasis in mouse models of MMTV-PyMT mammary cancer, breast cancer with nicotine exposure and melanoma, all of which are closely associated with the occurrence of pulmonary metastasis [79, 126, 127]. Neutrophils also contribute to ovarian cancer metastasis to the omentum by premetastic niche formation [128]. In cancer-bearing mice, cancer tissues modulate the microenvironment in the distal organ by releasing various cytokines, including vascular endothelial growth factor A (VEGFA), TNF, transforming growth factor-β (TGFβ), and G-CSF, to prepare for subsequent cancer metastasis [126, 129]. Blockage of neutrophil recruitment to the premetastatic sites or NET formation often prevents metastasis. However, whether targeting this phenomenon can prevent cancer metastasis into other organs or tissues, which are common as metastatic sites such as brain, breast, and lymph nodes, remains to be further investigated.

### Neutrophils inhibit cancer metastasis

In contrast, other researchers have shown that neutrophil depletion facilitates metastasis. CCL2 and G-CSF secreted by the primary tumor activate the cytotoxic functions of these antimetastatic neutrophils mediated by H_2_O_2_. The type of tumor-entrained neutrophils is only observed in patients with cancer and not in healthy people; neutrophils migrate from primary breast tumor sites into the lung before metastatic cancer cells and then exert an inhibitory effect on metastatic colonization [130]. Neutrophils produce chemokines that recruit T cells and other leukocytes to indirectly kill cancer cells [131]. In a mouse model of breast cancer cell metastasis to the lung, the inhibitory effect of neutrophils required the presence of NK cells. In the absence of NK cells, the tumoricidal activity of neutrophils switched into metastatic facilitation [132]. Moreover, neutrophil expression of thrombospondin 1, IL-1β and the receptor tyrosine-protein kinase MET limit the formation of metastases by blocking the cancer cell mesenchymal-to-epithelial transition and releasing NO individually [69, 105, 133]. Neutrophils acquire the characteristics of antigen-presenting cells (APCs) in the early stage and thus might stimulate the proliferation of T cells to protect against tumor metastasis [134].

## Neutrophils in cancer recurrence

According to clinical data, the NLR predicts the prognostic outcome and the absolute neutrophil counts are considered independent prognostic factors for cervical cancer relapse and postoperative recurrence of intrahepatic cholangiocarcinoma [135, 136]. Although the underlying mechanism remains unclear, the interaction between neutrophils and cancer cells may play a role in cancer recurrence. In a zebrafish melanoma model, neutrophils were recruited to the inflammatory site of postoperative trauma and interacted with precancerous cells, providing them with environmental conditions that support their proliferation, and these interactions may be associated with postoperative cancer relapse. In ovarian and lung cancer, stress hormone-induced neutrophil activation reactivates dormant cancer cells and leads to early recurrence. Neutrophil activation is based on the release of S100A8/A9 proteins, myeloperoxidase activation and oxidized lipid accumulation, which finally activate the fibroblast growth factor-related signaling pathway in dormant cancer cells and push them to exit from dormancy [76]. In patients with breast cancer diagnosed with COVID-19, emerging reports show that dormant cancer cells are reawakened by factors released during lung inflammation, including NETs. Severe acute respiratory syndrome coronavirus 2 infection of airway epithelial cells first releases damage-associated molecular patterns followed by inflammatory cytokines and chemokines, which further recruit and activate neutrophils to release NETs [137].

Taken together, these findings show that the premetastatic behavior of neutrophils can be switched in vivo, providing possible opportunities for therapeutic intervention (Table 2). Although cancer recurrence is currently proposed to increase in the presence of neutrophils, our understanding of the role of neutrophils might be altered as this field advances.

**Table 2:** Neutrophil roles in carcinogenesis, cancer growth and metastasis

Cancer	Year	Species	Mechanism	References
*Cancer-promoting role*				
Lung cancer	2010	Mouse	Neutrophil elastase accelerates lung cancer growth via degradation of IRS-1	[138]
Lung carcinoma, melanoma	2016	Mouse	NETosis promotes cancer growth	[139]
Small intestinal cancer	2016	Mouse	Hypercoagulation induced by NETosis promotes carcinogenesis and N2 polarization	[140]
Lung adenocarcinoma	2017	Human, mouse	A distinct subset of SiglecFhigh neutrophils dependent on cancer-induced osteoblastic cells promote cancer growth	[141]
Melanoma	2017	Mouse	Neutrophils recruit to TME and acquire immunosuppressive properties	[142]
Lymphoma, Lung carcinoma, colon carcinoma, pancreatic cancer	2019	Mouse	Neutrophils acquire immunosuppressive activity mediated by FATP2	[143]
Lung adenocarcinoma	2020	Human	Multi-omics reveal a potential immunosuppressive role of neutrophil degranulation	[144]
Hepatocellular carcinoma	2011	Human	Neutrophil is correlated with angiogenesis progression	[145]
Pancreatic cancer	2016	Mouse	CXCR2 signaling promotes carcinogenesis and metastasis	[146]
Lung carcinoma	2013	Mouse	NETs trap circulating cancer cells and promote metastasis	[121]
Breast cancer	2015	Mouse	Neutrophil-derived leukotrienes establish the lung pre-metastatic niche	[126]
Breast cancer	2015	Mouse	Neutrophils polarized by IL-17-producing γδ T cells acquire the ability to suppress cytotoxic T lymphocytes and promotes metastasis	[113]
Lung carcinoma, melanoma	2016	Mouse	Neutrophils recruited by TLR3 promote lung pre-metastatic niche formation	[127]
Breast cancer	2016	Mouse	NETs induced by cancer promote metastasis	[147]
Breast cancer	2018	Mouse	NETs produced during inflammation awaken dormant cancer cells	[72]
Breast cancer	2019	Mouse	WNT-dependent systemic neutrophilic inflammation triggered by loss of p53 in cancer cells promotes metastasis	[148]
Breast cancer	2019	Human, mouse	Neutrophils escorting CTCs drives cell cycle progression and expands the metastatic potential of CTCs	[123]
Breast cancer, colon cancer	2020	Human	NETs promote metastasis via binding CCDC25 on cancer cells	[149]
*Cancer-suppressing role*				
Breast cancer	2011	Mouse	Neutrophils inhibit lung metastasis by generating H_2_O_2_	[130]
Lung cancer	2014	Human	TANs stimulate T cell responses in the early stage of lung cancer	[150]
Uterine cancer	2015	Mouse	Neutrophils oppose carcinogenesis via clearance of hypoxic cancer cells	[151]
Lung cancer	2016	Human	TANs act as APCs in early-stage lung cancer	[152]
Colorectal cancer	2017	Human	Neutrophils enhance the responsiveness of CD8^+^ T cells and improve survival	[153]
Undifferentiated pleomorphic sarcoma (UPS)	2019	Human	Neutrophils driving UTCαβ polarization and type 1 immunity mediate resistance against UPS	[154]
Uterine cancer	2020	Mouse	Neutrophils kill cancer cells via their production of ROS and MMP-9 upon relief of hypoxia	[155]
35 cancer cell lines	2021	Human	Neutrophil elastase selectively kills cancer cells and attenuates carcinogenesis	[70]
*Neutrophil-associated complications in cancer*				
Mammary carcinoma	2015	Mouse	Kidney and heart failure caused by NETosis and inflammation	[156]
Lung carcinoma	2015	Mouse	HGF/MET-dependent neutrophil recruitment and NO release by neutrophils promotes cancer cell killing	[105]
Small intestinal cancer	2016	Mouse	Coagulation promoted by NETosis	[140]
Myeloproliferative neoplasm	2018	Mouse	Thrombosis promoted by increased NETosis	[157]
*Neutrophils with anticancer therapeutic role*				
Non-Hodgkin lymphoma	2010	Mouse	Neutrophils kill cancer cells by phagocytosis in the treatment of anti-CD47 antibodies synergized with rituximab	[158]
Thymoma, breast cancer	2010 2014	Mouse	MDSCs are selectively killed by 5-Fluorouracil or doxorubicin selectively resulting in enhanced T cell-dependent anticancer immunity	[159, 160]
Different cancer cell lines	2013	Mouse	5-FU and gemcitabine can promote cancer inflammation and resistance to chemotherapy mediated by neutrophils and T cells	[161]
Different cancer cell lines	2016	Mouse	Radiotherapy induces infiltration of neutrophils with cytotoxic activity against cancer cells	[162]
Glioma	2017	Mouse	Neutrophil can act as a vector of anticancer drug delivery to cross BBB for suppression of postoperative malignant cancer recurrence	[163]
Different cancer cell lines	2018	Mouse	Neutrophils kill antibody-opsonized cancer cells by trogoptosis	[107]
Lung carcinoma	2018	Mouse	TANs are reprogrammed to promote anticancer immunity by blocking LILRB2	[164]
Different cancer cell lines	2020	Mouse	Neutrophils kill cancer cells via ADCC mediated by IgA and enhanced by CD47–SIRPα checkpoint inhibition	[165]

## Neutrophil plasticity and the cancer microenvironment

### Cancer microenvironment mediates dual roles of neutrophils

In cancer, neutrophils exert both pro-cancer and anticancer effects. The diversity of neutrophils is very common in cancer. A transcriptomic analysis revealed that tumor-associated neutrophils (TANs) and neutrophils from patients with cancer or cancer-bearing mice, which showed a higher proportion of neutrophil progenitors and a tendency toward immunosuppressive properties, differed significantly from those from healthy people or mice [166]. This diversity results from the high plasticity of neutrophils due to the effects of complex cancer microenvironments. The cancer and tissue microenvironments, conventional therapies and immunotherapy shape neutrophil function.

In a GEMM of lung adenocarcinoma, TGFβ polarized neutrophils in a cancer-promoting direction, and TGFβ blockade reversed the neutrophil protumor phenotype to an antitumor phenotype. These two types of neutrophils with opposite functions are named N2 and N1, respectively, which are similar and comparable to tumor-associated macrophages, such as M2 and M1 [167]. In the early stage of non-small cell lung cancer, the anticancer state of neutrophils is also induced by interferon-γ (IFNγ) and GM-CSF. Induced neutrophils indeed develop from immature progenitors through the negative regulation of the transcription factor Ikaros and acquire APCs properties, which as APC-like hybrid cells, promote T cell antitumor responses [152]. Another study has shown that hypoxia is a potent determinant of the TAN phenotype and direct neutrophil-cancer cell interactions. After the removal of hypoxia, the number of neutrophils recruited by the cancer decreased significantly, but the recruited cells were more effective at killing the cancer cells. This activity is mediated by the production of NADPH oxidase-derived ROS and MMP-9. At the same time, the ability of neutrophils to promote cancer cell proliferation, which appears to be mediated by their production of NE, is also reduced [155]. The general trend is that TANs belong to a network of anticancer cells in the early stages of carcinogenesis, but with cancer progression, neutrophil function shifts to immunosuppressive and cancer-promoting states.

### Metabolic reprogramming of neutrophils

Neutrophils among TANs with proven immunosuppressive function have been extensively studied and have been named granulocytic myeloid-derived suppressor cells (G-MDSCs) or polymorphonuclear myeloid-derived suppressive cells. G-MDSCs appear as neutrophils at different stages of maturation [168]. G-MDSCs flexibly adapt to the cancer microenvironment. The most important of these adaptations is the metabolic shift, which exerts a substantial effect on cell function.

Metabolic features include the upregulation of fatty acid transport protein 2 (FATP2) [143], increased levels of arginase I [169], high NADPH oxidase activity [155] and active NOS [170]. These factors have all been shown to inhibit T cell function. Regarding the accumulation of high lipids in cancer microenvironment, G-MDSCs increase the uptake of exogenous fatty acids through STAT3- or STAT5-mediated upregulation of lipid transport receptors. Increased fatty acid oxidation induces G-MDSCs to undergo metabolic reprogramming from glycolysis and become immunosuppressive [171]. Accordingly, inhibition of the neutrophil metabolic reprogramming by blocking fatty acid oxidation can synergize with the immunotherapeutic effect of T cells. Thus, neutrophils not only utilize diverse metabolic strategies to meet the energy requirements for survival but also exhibit functional alterations in cancer based on changes in the cancer microenvironment, such as decreased glucose levels, low oxygen pressure and low pH values [172]. Cancer can produce many factors such as IL-1β, CCL2, TGF-β, G-CSF and GM-CSF influencing innate immune cells, including neutrophils [173]. In particular, G-CSF, GM-CSF and IL-6 secreted by cancer and/or by stromal cells surrounding cancer cells induce potent activation of G-MDCs by activating the myeloid transcription factor C/EBPβ.

### Neutrophil subset identification and markers

Researchers have attempted to identify neutrophil subsets. Specific surface markers proposed to identify neutrophil subsets in cancer include CD101 and CD177 [174, 175], which are associated with cancer regression, and CD117, PDL1, CD170, LOX1, CD84 and JAML [176], which are associated with T cell immunosuppression and cancer progression. In PDAC, the purinergic receptor P2RX-negative neutrophil subset exhibits immunosuppressive role with enhanced PD-L1 expression and mitochondrial metabolism [177]. However, an unequivocal method to detect immunosuppressive neutrophils and other neutrophil subsets using flow cytometry or other strategies remains to be developed. Since the subsets of neutrophils show continuous changes and are highly phenotypically and morphologically similar (even between MDSCs and other cells), a reasonable assumption is that these hypothetical subsets are actually the same type of cells, with larger or smaller changes induced by different local environments. These neutrophils are a single cell type with many different functional phenotypes. The high plasticity of neutrophils enables them to respond quickly to external stimuli, leading to their heterogeneity. Because different stimuli mobilize different cytoplasmic granules, different degrees of exposure of the membrane proteins of each granule to the cell surface can change the cell surface composition of neutrophils, potentially leading to the misidentification of new cell types. Taken together, TANs appear to be more flexible than circulating neutrophils, which enables them to adapt to diverse cancer microenvironments.

Moreover, TANs or normal neutrophils have consistently been shown to be trained to become anticancer neutrophils through various methods to achieve the goal of killing cancer cells. For example, PPM1D/Wip1 is a negative regulator of the cancer suppressor p53 and is overexpressed in several human solid cancers. *Ppm1d* knockout or chemical inhibition of Wip1 in human or mouse neutrophils exacerbates anticancer phenotypes and increases p53-dependent expression of costimulatory ligands and the proliferation of cocultured cytotoxic T cells [178]. Another study showed that exposure to β-glucan [179], a fungal-derived prototype agonist of trained immunity, trained neutrophils in mice to enhance the anticancer activity of neutrophils. These results, in turn, prove that neutrophils are highly plastic (Fig. 1C).

### Interaction between neutrophils and other microenvironmental cells

Cancer is highly heterogeneous and is considered one of its hallmarks. The tumor contains cancer cells and noncancerous cells such as neutrophils, macrophages, T cells, adipocytes, stromal cells and others constituting the microenvironment. All these cells communicate directly or indirectly. Thus, neutrophils in cancer not only have a relationship with the T cells mentioned above but also affect or are affected by other cells. During advanced colorectal cancer progression, cancer stem cell-derived exosomes containing triphosphate RNAs prime neutrophils for cancer development and depletion of neutrophils with antibodies attenuate the tumorigenicity of these cancer stem cells [180]. In obese patients with pancreatic cancer, crosstalk among pancreatic stellate cells, neutrophils and adipocytes mediated by IL1β promotes PDAC. Genetic or pharmacological targeting of this circuit provides a potential method for pancreatic cancer treatment [181]. Cancer-associated fibroblasts are considered one of the important stromal cells contributing to cancer development. A recent report identified that one of the underlying mechanisms as NET induction. This induction is driven by increased amyloid and β-secretase expression in fibroblasts [182].

## Discussion and perspectives

We speculate that the cancer microenvironment may reprogram neutrophils to achieve conversion between anticancer polarity and cancer-promoting one. First, as previously described, neutrophils are heterogeneous in patients with cancer, which may result from the reprogramming of mature neutrophils. Many data indicate that neutrophil precursors support cancer growth and metastatic progression. Second, cancer cells functionally shape the cancer microenvironment by secreting various cytokines, chemokines and other factors, which provides the necessary environmental conditions for the reprogramming of surrounding neutrophils. Neutrophils acquiring new transcriptional activity, which could be characterized as diverse neutrophil subsets, based on single cell RNA sequencing analysis under specific microenvironment support the hypothesis [183]. Our previous review also stated that cancer cells undergo cellular reprogramming either spontaneously or after anticancer treatment [184]. All of these findings suggest the possibility of reprogramming both cancer cells and neutrophils in the cancer microenvironment. Third, our experiments show that mature neutrophils are reprogrammed into multipotent progenitors in the presence of a chemical cocktail [185]. In other words, neutrophils have the potential to undergo cell reprogramming.

More evidence of neutrophil reprogramming is illustrated below. Neutrophils transdifferentiate into other cell types. One study has shown that human postmitotic neutrophils are reprogrammed into macrophages via growth factors. The molecular mechanisms underlying functional changes in neutrophils has been discovered that GM-CSF controls the overexpression of FATP2 in neutrophils through the activation of the STAT5 transcription factor, thereby enabling neutrophils to obtain immunosuppressive activity and promote cancer progression in mice [143]. In addition, metabolic reprogramming of neutrophils leads to functional changes, as a metabolic shift of innate immune cells, including neutrophils, is observed in pulmonary diseases, accompanied by an impaired normal immune function of these cells.

In conclusion, neutrophils exert both pro-cancer and anticancer effects on the initiation, growth and metastasis of cancer, and these different functions are accompanied by the existence of different neutrophil subpopulations. Because neutrophils normally possess antimicrobial and anticancer functions, functional transformation or abnormal cell differentiation must occur. Here, we propose a hypothesis that the cancer microenvironment or clinical treatment may induce the reprogramming of neutrophils. In clinical practice, an elevated NLR serves as a prognostic indicator and the inhibition or reversal of neutrophil reprogramming can also be employed as a potential therapeutic strategy, *e.g.*, conversion of neutrophils into antigen-presenting cells by FcγR engagement can exhibit immunotherapeutic effect on cancer [186].

## Conclusions

Neutrophils would be a promising cell target population for anticancer therapy, although their roles in cancer are dual and remain to be further investigated. Direct target neutrophils or indirect target microenvironment factors reprogramming neutrophil plasticity might be potential therapeutic strategies.

## Data Availability

Not applicable.

## Abbreviations

ADCC: Antibody-dependent cellular cytotoxicity
COVID-19: Coronavirus disease 2019
CTCs: Circulating cancer cells
FATP2: Fatty acid transport protein 2
GEMMs: Genetically engineered mouse models
G-MDSCs: Granulocytic myeloid-derived suppressor cells
GMP: Granulocyte–monocyte myeloid progenitor
HGF: Hepatocyte growth factor
HMGB1: High mobility group protein B1
HOCl: Hypochlorous acid
HPRT: Hypoxanthine phosphoribosyl transferase
IFNγ: Interferon-γ
IL-1RA: IL-1 receptor antagonist
iNOS: Inducible nitric oxide synthase
LMPPs: Lymphoid-primed multipotent progenitors
MPO: Myeloperoxidase
NE: Neutrophil elastase
NETs: Neutrophil extracellular traps
NK: Natural killer
NLR: Neutrophil-to-lymphocyte ratio
NOS: Nitric oxide synthase
PDAC: Pancreatic ductal adenocarcinoma
proNeu1: Early committed neutrophil progenitor
proNeu2: Intermediate progeny
preNeu: Preneutrophil
RNS: Reactive nitrogen species
ROS: Reactive oxygen species
TANs: Tumor-associated neutrophils
TGFβ: Transforming growth factor-β
TNF: Tumor necrosis factor
VEGF: Vascular endothelial growth factor
